# Measurement matters for assessing the role of chronically altered perfusion in post-stroke aphasia

**DOI:** 10.1093/braincomms/fcad341

**Published:** 2023-12-08

**Authors:** Cynthia K Thompson, Matthew Walenski

**Affiliations:** Department of Communication Sciences and Disorders, Northwestern University, Evanston, IL, USA; Cognitive Neurology and Alzheimer’s Disease Center, Northwestern University, Evanston, IL, USA; Department of Communication Sciences and Disorders, East Carolina University, Greenville, NC, USA

## Abstract

This scientific commentary refers to ‘Cerebral perfusion in post-stroke aphasia and its relationship to residual language abilities’, by Ivanova *et al*. (https://doi.org/10.1093/braincomms/fcad252).

**This scientific commentary refers to ‘Cerebral perfusion in post-stroke aphasia and its relationship to residual language abilities’, by Ivanova *et al*. (https://doi.org/10.1093/braincomms/fcad252).**

The impact of stroke-induced physiological changes in brain function on recovery from stroke is important for understanding and predicting recovery trajectories. Reduced cerebral blood flow (hypoperfusion), one such physiological change, is commonly observed in acute stages of recovery^1^ and has recently been shown to persist into the chronic stage of recovery, long after stroke.^2,3^ In their recent article in *Brain Communications*, Ivanova *et al*.^4^ investigated cerebral perfusion in 43 individuals with chronic aphasia resulting from stroke and found decreased perfusion values in frontal and parietal regions in the left (stroke-lesioned) hemisphere, a result largely supporting previous studies, including our 2017 study with 35 chronic aphasic participants, although, our region-of-interest (ROI) analyses found hypoperfused tissue only in left posterior (primarily temporoparietal) regions.^5^

Physiologically, it is also clear that surviving tissue near to the stroke lesion remains hypoperfused many months or years after the stroke. Several research groups have studied perfusion values in perilesional regions, defined as rings of tissue around a lesion (hereafter referred to as ‘circumlesional’). Ivanova *et al*.^4^ found reduced perfusion values in left hemisphere circumlesional tissue, with the tissue closest to the lesion the most hypoperfused. This finding also replicated the results of our 2017 study^5^ as well as our 2022 study, in which we found this pattern in 71 participants with chronic aphasia.^6^

The question is: to what extent does change in cerebral blood flow account for impairments in language in people with aphasia? Since blood supply is required for the brain to function, a correlation between perfusion and function can be hypothesized. Importantly, however, the findings reported by Ivanova *et al*.^4^ differ from those derived from our studies. Ivanova *et al*.^4^ found that performance on language measures was positively correlated with perfusion levels in left parietal, but not frontal, ROIs. In addition, they found a marginal relation between perfusion values in left circumlesional tissue and language ability. Conversely, we found no relation between perfusion values and language abilities across ROIs in the left hemisphere.^5^ We also found no relation between language ability and left (or right) hemisphere circumlesional perfusion in participants with chronic aphasia, either prior to or following (successful) domain-specific language treatment.^6^ At pre-treatment, participants in both treatment and control groups showed abnormally decreased blood flow across left hemisphere circumlesional regions and abnormally increased blood flow in right hemisphere rings mirroring those in the left, which was unchanged on follow-up testing 3 months later for both groups, even though participants in the treatment, but not the control, group showed significantly improved language.

One issue related to this lack of agreement across studies pertains to how language functions were measured. Ivanonva *et al*.^4^ relied on the Western Aphasia Battery (WAB) to provide an index of language function in their participants. While the WAB is perfectly well suited to detect broadly impaired language, it lacks the detail and linguistic specificity that more specialized instruments can provide. The choice of the WAB may therefore obscure efforts to relate perfusion in (potentially highly) specialized language regions to their (potentially highly) specialized language function. In any case, the use of different language assessment instruments across studies complicates the comparison of results across studies. This complication may ease over time, as the field develops a greater understanding of the functional roles of particular brain regions and the best instruments to use to detect spared/impaired language function in those regions.

Another discrepancy between the results reported by Ivanova *et al*.^4^ and previous studies concerns the impact of left hemisphere stroke on right hemisphere cerebral blood flow. Ivanona *et al*.^4^ found no difference between patients and healthy controls in the right hemisphere, whereas we found that perfusion values in both frontal and parietal–occipital regions in the right hemisphere were greater (hyperperfused) in people with aphasia.^5^ In addition, we found this hyperperfusion pattern in right hemisphere cortical rings mirroring left hemisphere circumlesional rings, and in right hemisphere lesion homologue regions, in our 71 participants with chronic aphasia.^5,6^ That is, the patients showed increased blood flow when compared with healthy controls in right hemisphere regions.

Methods used to normalize the raw perfusion values, to control for individual variation in blood flow (which may reflect a variety of factors)^7^, may explain these differences in results across studies. In our previous work,^5,6^ we chose perfusion values in the right occipital cortex as the standard of comparison for normalization. We chose this region because of its location distal to the lesions in our sample and because it is supplied by the posterior cerebral artery that was ostensibly unaffected by the stroke lesions in our participants. Of course, occipital perfusion may not be entirely ‘normal’, as the conditions that precipitated the strokes in our participants may have had some impact on vascular tissue broadly, but that contributes to the need to normalize the raw perfusion values in the first place. Of particular importance, however, is that the occipital region did not include either lesioned tissue or circumlesional tissue. The resulting values are straightforward to interpret—normalized perfusion values close to 1 are ‘normal’, values <1 are hypoperfused and values >1 are hyperperfused. This is also relatively easy to compare across studies—with the caveat that studies may need to choose different regions as their standard, depending on lesion location and size within a sample.

In contrast, Ivanova *et al*.^4^ normalized their perfusion values with the average perfusion value of the full brain as the standard, excluding only the lesion itself. Thus, their standard necessarily included the hypoperfused circumlesional tissue, and more of it for larger compared with smaller lesions. A potential benefit of this choice is that the normalized values are straightforward to compare across studies, since the whole brain (barring variably located lesions) is a common standard, avoiding potential difficulties related to choosing different regions for the standard across studies. However, the impact of this choice is that normalized perfusion values in hypoperfused regions will be closer to 1 than they perhaps should be, and ‘normally’ perfused regions may appear to be hyperperfused. This could easily confound analyses relating perfusion levels to language function. This impact is not directly undone by including lesion size as a covariate in a further analysis (as Ivanova *et al*. did). As the authors note, the impact of these choices on the correspondence between perfusion levels and behaviour needs further investigation.

Despite differences across studies, a final question relevant to studying perfusion values in post-stroke aphasia is: does altered blood flow in critical cortical regions within the left or right hemisphere impact the extent to which these regions can be recruited into the language network to support language recovery? Reasonable hypotheses are that neuroplasticity (i.e. the takeover of function by new brain regions) is precluded in regions where blood flow is decreased (hypoperfused), rendering it physiologically incapable of supporting function in the left hemisphere or enhanced in right hemisphere regions where blood flow is increased (hyperperfused). This question, to our knowledge, has not been directly addressed, but is important for understanding how change in perfusion affects language recovery in people with aphasia.

In sum, the present paper by Ivanova *et al*.^4^ offers a welcome addition to the literature on perfusion-function correspondences in aphasia. We hope this brief commentary serves to highlight important converging findings derived across published studies as well as divergent findings and potential methodological reasons for them. Future work in the area should continue to address the relation between post-stroke abnormal perfusion and language impairments using specialized language measures to chart the relation between perfusion in critical brain regions and linguistic impairments. Research focused on the extent to which altered perfusion values in critical brain regions either precludes or possibly enhances neuroplasticity also is needed. Finally, future work in this area must carefully consider methods used to normalize perfusion values, avoiding using cortical regions in which perfusion is directly impacted by stroke (i.e. whole brain analyses that include circumlesional tissue).
